# Assessing the Potential Distribution of the Traditional Chinese Medicinal Plant *Spatholobus suberectus* in China Under Climate Change: A Biomod2 Ensemble Model-Based Study

**DOI:** 10.3390/biology14081071

**Published:** 2025-08-17

**Authors:** Yijun Lin, Quanwei Liu, Shan Lv, Xiaoyu Huang, Chaoyang Wei, Jun Li, Yijie Guan, Yaxuan Pan, Yijia Mi, Yanshu Cheng, Xiangyu Yang, Danping Xu

**Affiliations:** College of Life Science, China West Normal University, Nanchong 637002, China; yijun6644@foxmail.com (Y.L.); quanwei66977@foxmail.com (Q.L.);

**Keywords:** *Spatholobus suberectus*, ensemble model, climate change, potential suitable habitat, distribution changes

## Abstract

*Spatholobus suberectus*, a valuable Chinese medicinal plant, faces habitat shifts under climate change. In order to better utilize the medicinal properties of *S. suberectus* and conduct further investigations, this study utilized the Biomod2 ensemble model to predict and analyze the potential expansion and contraction of suitable habitat areas for *S. suberectus* in China under changing climatic and environmental conditions. The results showed that, compared to the pre-screened models, the ensemble model significantly improved the prediction accuracy. Currently, *S. suberectus* is primarily distributed in southern China. Under the projected scenarios of SSP1-2.6, SSP2-4.5, and SSP5-8.5, its suitable habitat is expected to expand overall, with the increased areas concentrated mainly in southwestern, central, and eastern China. As climatic factors shift, the high-suitability center of *S. suberectus* is predicted to shift slightly southward under the SSP1-2.6 scenario, while under the SSP2-4.5 and SSP5-8.5 scenarios, it is projected to move northwestward. In the future, it will be necessary to optimize the warm and humid growth environment for cultivated *S. suberectus* in China. Meanwhile, wild *S. suberectus* populations should be closely monitored for the impact of climate change to prevent potential partial reductions in suitable habitats, thereby ensuring ecological balance and sustainable development.

## Simple Summary

In order to make better use of the medicinal value of *Spatholobus suberectus* and further investigate it, the Biomod2 ensemble model was used to predict the future trend of the suitable area of *S. suberectus*. At present, the suitable area of *S. suberectus* is mainly distributed in southern China, and with the change in various climatic factors, the suitable areas of *S. suberectus* will expand as a whole in the future. This study can provide a reference for the planting of *S. suberectus* in China, such as giving priority to semi-shady slope or forest edge areas for artificial planting.

## 1. Introduction

*Spatholobus suberectus (Leguminosae)*, “Ji-xue-teng” in China, is a climbing vine plant [1]. The flower of this plant is conical inflorescence, axillary or at the top of small branches, with short yellow-brown hairs on the inflorescence and peduncle. The corolla is white, the flag is flattened and round, the wing petals are slightly longer than the keel petals, the anthers are spherical in shape and uniform in size, and the ovary is sessile and covered with rough hairs. The fruit is pod-shaped, knife-shaped, and densely covered with short brown hairs and has a fruit neck. The seeds are oblong and flattened [2]. *S. suberectus* is mainly distributed in southern China (such as Yunnan, Guangxi, and Guangdong), and mostly grows in sparse forests, dense forests, valleys, or shrubs at an altitude of 800–1700 m [3].

*S. suberectus* is a widely used traditional Chinese medicine. It has a bitter taste, a sweet flavor, and primarily acts on the liver and kidney meridians. Research has shown that *S. suberectus* has significant clinical effects, such as inhibiting the growth of human breast cancer cells [4] and human lung adenocarcinoma cells (anticancer) [5]. Its flavonoid extracts also exhibit antioxidant activity, with certain compounds capable of scavenging free radicals. Additionally, it has protective effects on hematopoietic function impaired by radiation, contributing to the improvement of the hematopoietic system. Furthermore, it has immunomodulatory, antiviral [6,7,8], blood-enriching, and meridian-activating properties, making it useful in the treatment of conditions such as menstrual irregularities, blood deficiency, numbness, paralysis, and rheumatic pain [9,10,11]. The effective chemical components are closely related to the pharmacological effects, with modern pharmacological studies identifying several bioactive compounds, such as isorhamnetin, lupeol, and dandelion sesquiterpene [12]. Among these, isorhamnetin has been shown to inhibit the proliferation of various tumor cells in vitro and significantly suppress tumor growth in vivo [13]. Lupeol alleviates postprandial hyperglycemia [14], and dandelion sesquiterpene inhibits cancer cell growth by inducing apoptosis [15]. Therefore, *S. suberectus* exhibits both anticancer and antidiabetic activities. At present, research on *S. suberectus* mainly focuses on its chemical composition and clinical efficacy, while studies regarding its geographic distribution are scarce. There is significant research potential in predicting the future potential distribution of *S. suberectus* in China under the influence of climate and other environmental data changes over the coming decades.

Global climate change is progressing rapidly [16], and environmental changes have been shown to affect species distribution. For example, the southern latitudinal boundary of South African mangroves is predicted to shift southward due to multiple environmental factors [17]. In Mexico, the suitable habitat area for *Tagetes lucida Cav.* (Asteraceae) is expected to migrate northward and decrease in size due to significant climate change [18]. As such, research predicting species’ responses to future environmental scenarios is necessary [19], and species distribution models (SDMs) have emerged as a tool for this purpose. SDMs are mathematical models that estimate species’ niche requirements based on distribution data and environmental variables, thus predicting potential species distributions and assessing the contribution of environmental variables. These models have been widely used in habitat suitability assessments, biological invasion risk evaluations, and species distribution change studies [20]. Various SDMs are available, including the Maximum Entropy Model (MaxEnt) [21], CLIMEX [22], Ecological Niche Factor Analysis (ENFA) [23], and others. The Biomod2 platform, based on R language [24], integrates 10 commonly used SDMs, including the Generalized Linear Model (GLM) [25], Generalized Boosted Regression Model (GBM) [26], Generalized Additive Model (GAM) [25], Classification Tree Analysis (CTA) [27], Artificial Neural Network (ANN) [28], Surface Range Envelope (SRE) [29], Flexible Discriminant Analysis (FDA) [30], Multivariate Adaptive Regression Splines (MARS) [31], Random Forest (RF) [32], and MaxEnt [21]. This platform allows for comprehensive species evaluation and integrates the best-performing models to predict species distributions, thereby maximizing prediction accuracy and enhancing the reliability of future distribution forecasts. It is currently one of the most mature multi-model platforms [27,33]. Ensemble models, which combine results from multiple models, have been shown to offer certain advantages [27,33,34]. Although research using ensemble models remains relatively limited [35], it is anticipated that future studies will increasingly adopt this approach.

Currently, there have been no reported studies on the distribution prediction of *S. suberectus*, nor have the factors affecting its distribution in China been extensively explored. Additionally, there is a lack of research predicting the future distribution of *S. suberectus* in China under climate change scenarios. Species distribution predictions can reflect changes in species’ adaptability to specific regions, the risk of overexploitation, and evolutionary trends [36]. The absence of such studies on *S. suberectus* could hinder efforts to monitor its exploitation rate and limit insights into its evolutionary patterns. Therefore, this study utilizes the Biomod2 ensemble model to predict the suitable habitat areas of *S. suberectus* in China over different time periods, aiming to analyze the changes in suitable areas under various climatic conditions, assess the migration trends of *S. suberectus* under climate change, and identify the key drivers influencing its distribution. This study aims to fill the gap in the distribution prediction of *S. suberectus*.

## 2. Materials and Methods

### 2.1. Acquisition and Screening of Species Distribution Data

**Figure 1:**
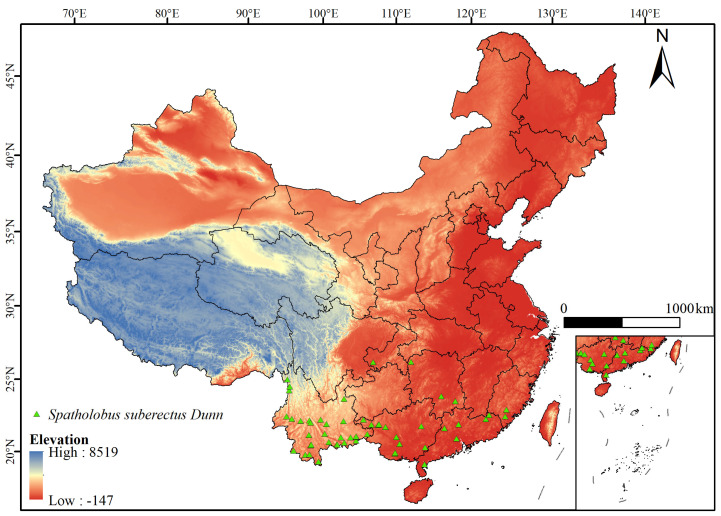
Distribution of *Spatholobus suberectus* in China (the green triangular points in the figure represent the coordinates of the current distribution points of *Spatholobus suberectus*).

The species distribution point coordinates of *S. suberectus* in China used in this study were primarily sourced from the following: (1) the China Digital Herbarium (https://www.cvh.ac.cn/, accessed on 7 August 2024); (2) the Global Biodiversity Information Facility (GBIF, https://doi.org/10.15468/dl.hy9gcv, accessed on 7 August 2024); and (3) literature retrieval (https://www.x-mol.com/, accessed on 7 August 2024). The obtained distribution data were imported into ArcGIS, and unreasonable distribution points were excluded based on relevant literature. A total of 118 distribution points of *S. suberectus* in China were initially compiled. To avoid model overfitting, the ENMTool was used to remove redundant data and verify and remove duplicate occurrence points based on spatial distance criteria, retaining only one distribution point within a 5 km radius; 118 distribution points were analyzed one by one [37]. Every time a point whose distance meets the redundancy standard was encountered, it was eliminated. After such a screening process, a large number of repeated or highly similar points in the spatial position were removed, and finally 53 distribution data which can more effectively represent the real distribution of *S. suberectus* in China without redundancy were obtained (Figure 1).

### 2.2. Acquisition and Screening of Environmental Data

This study utilized two sets of environmental data, comprising 19 climate variables, 11 soil variables, and 3 topographic factors, totaling 33 environmental variables. The climate data were obtained from the WorldClim database (https://www.worldclim.org/, accessed on 7 August 2024), soil quality data were sourced from the Global Soil Database (https://gaez.fao.org/pages/hwsd, accessed on 7 August 2024), and the Human Influence Index was derived from the Earth Observing System Data and Information System (EOSDIS) managed by NASA, in collaboration with the Socioeconomic Data and Applications Center (SEDAC) (https://sedac.ciesin.columbia.edu/, accessed on 7 August 2024). Combined with relevant data on factors influencing the distribution of *S. suberectus* in China, 11 soil variables were selected and extracted in ArcGIS.

**Figure A1:**
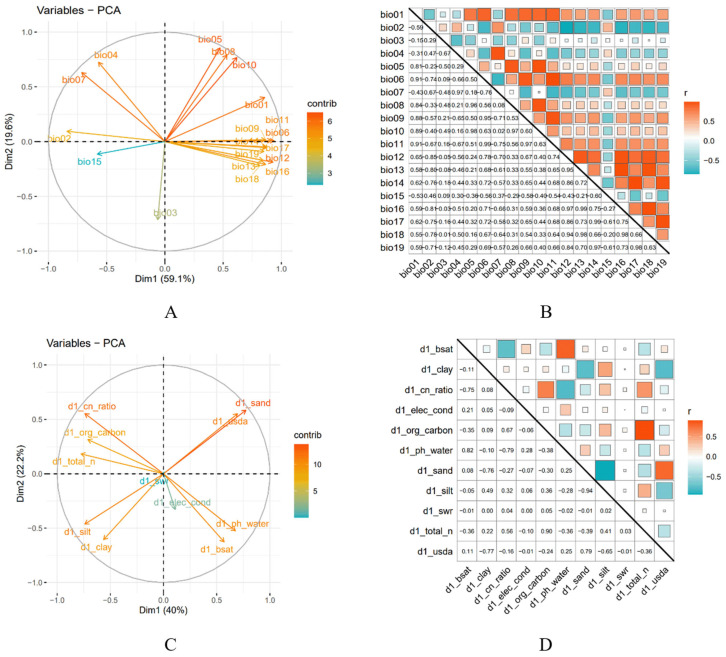
Principal component analysis (PCA) of 19 climates: (**A**) PCA of climate variables (PCA2); (**B**) heatmap of the correlation analysis of climate variables. (**C**) PCA of soil variables (PCA2), (**D**) Heatmap of the correlation analysis of soil variables.

The Biomod2 software was used to predict the potential geographical distribution of *Spatholobus suberectus* in China under current and future (2050s, 2070s, and 2090s) climate scenarios (SSP1-2.6, SSP2-4.5, and SSP5-8.5). Among them, the SSP1-2.6 scenario represents a combination of low emissions, low vulnerability, low mitigation pressure, and low radiative forcing; the SSP2-4.5 scenario represents an intermediate development pathway, a combination of moderate social vulnerability and moderate radiative forcing; and the SSP5-8.5 scenario represents a high-emission and high social development scenario. The selection of environmental variables is a crucial step in building a robust Biomod2 model. Therefore, a scientific screening process was applied: the extracted data were resampled in ArcGIS, and the resolution was standardized to 2.5’ (5 km × 5 km). The 19 bioclimatic factors and 11 soil variables were subjected to principal component analysis (PCA) and correlation heatmap analysis (Figure A1). Variables with high contributions in PCA1 and PCA2 and a correlation lower than 0.8 were selected. Subsequently, variables with correlations greater than 0.8 were removed, along with those based on soil variable PCA and correlation analysis heatmaps (Table 1).

### 2.3. Model Construction and Evaluation of Combined Model Accuracy

In this study, the Biomod2 package was used to model the distribution prediction of *S*. *suberectus* in China. Eleven different species prediction models, GLM, GBM, GAM, CTA, ANN, SRE, FDA, MARS, RF, MaxEnt, and XGBOOST, were applied from the Biomod2 package. To improve the accuracy of the model, the DISK method was used to create pseudo-absences. First, the locations of presence points of *Spatholobus suberectus* in China were determined based on the screened distribution points, and these data are represented by green triangles in the figures. Then, true absences were created according to the positions of the presence points and based on the geographical scope of China. Finally, when the number of true absence points was insufficient, three sets of pseudo-absence datasets (PA1, PA2, and PA3) were created at the boundary of the distribution range of *Spatholobus suberectus*, with 1000 points in each set. A random 25% of the distribution data of *Spatholobus suberectus* in China was selected for model testing, and 75% was used for model training. Each model was repeated 10 times, and a total of 300 model simulation results were finally established [38].

The contribution rates of each environmental factor to the model were comprehensively evaluated by the Biomod2 software package. The accuracy of the model was evaluated by both the True Skill Statistic (TSS) and Key Activity Performance Analysis (KAPPA) (Table 2). TSS is a test of sensitivity and specificity, and it is not affected by the size of the validation data, which can make up for the shortcomings of KAPPA in the evaluation process [39]. Both TSS and KAPPA are in the range of [0, 1], and the closer the results are to 1, the better the model construction effect. Models with TSS > 0.9 and KAPPA > 0.9 were selected to build the ensemble model and re-model, and the accuracy of the ensemble model was evaluated by TSS and KAPPA values. In this study, the results generated by the Biomod2 model were converted into raster data and visualized by using the Reclassify function of ArcGIS. The ensemble model obtained the results of suitable habitat probability values in the range of [0, 1]. Based on the cutoff value as the boundary, combined with the TSS threshold of the ensemble model, the habitat suitability index (HSI) and the physiological characteristics of the species, the potential suitable habitat areas were divided into four grades: unsuitable habitat area (0 ≤ *p* ≤ 0.263), low-suitability habitat area (0.263 < *p* ≤ 0.525), moderately suitable habitat area (0.525 < *p* ≤ 0.763), and highly suitable habitat area (0.763 < *p* ≤ 1) [40].

## 3. Results

### 3.1. Model Evaluation

**Figure 2:**
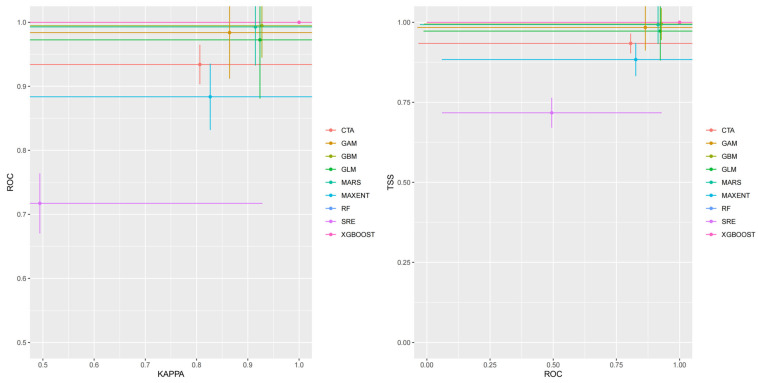
KAPPA–ROC curve and ROC–TSS curve of the ensemble model and individual models. The average Kappa value and TSS value of the RF model and the XGBOOST model are the highest, and the values are the same. Therefore, the two models in the figure are on the same horizontal line, which causes one of the graph lines to be blocked.

The Kappa value of the combined model was 0.908, and the TSS value was 0.916. Regarding the Biomod2 package, the RF model and XGBOOST model had the highest mean Kappa and TSS values. Although the mean values of KAPPA and TSS for the RF model and XGBOOST model are both higher than those of the combined model, the RF model or XGBOOST model, as a single model, has limitations in certain aspects. These results indicate that the combined model provided more accurate predictions of the geographic distribution of *S. suberectus*. Additionally, by integrating the predictions from multiple individual models, the combined model effectively reduced the potential fluctuations caused by the inherent limitations of any single model, thereby yielding more stable prediction results (Figure 2).

### 3.2. Selection of Environmental Factors and Contribution Rate Analysis

**Figure 3:**
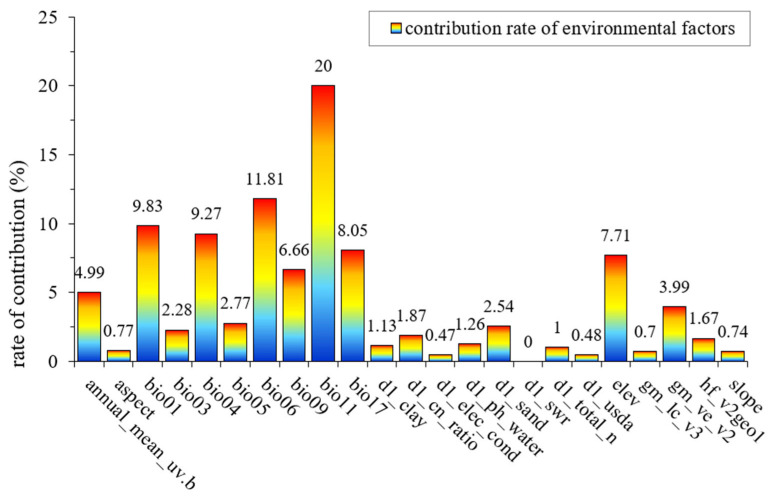
Contribution rates of environmental factors. The higher the bar chart, the greater the contribution rate.

Based on the correlation analysis between environmental factors and the contribution rate of each factor to the model, the percentage contribution of each environmental factor was obtained. This study focused on the five environmental factors with the highest contribution rates for further discussion (Figure 3). The results indicated that the top five environmental factors in terms of contribution rate were as follows: the average temperature of the coldest quarter (bio11), with a contribution rate of 20%; the minimum temperature of the coldest month (bio06), with a contribution rate of 11.81%; the annual average temperature (bio01), with a contribution rate of 9.83%; temperature seasonality (bio04), with a contribution rate of 9.27%; and the precipitation of the driest season (bio17), with a contribution rate of 8.05%.

**Figure A2:**
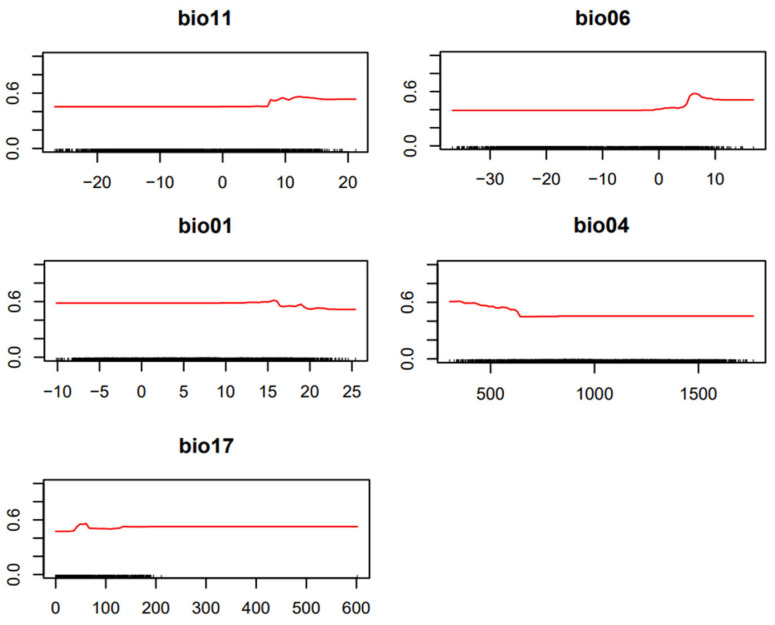
Influence curves of environmental factors.

While determining the environmental variables, the combined model also analyzed the response curves of *S. suberectus* to environmental factors (Figure A2). The adaptation range of *S. suberectus* to each environmental variable was roughly identified: the average temperature of the coldest quarter was around 8–14 °C, with an optimal value at approximately 12 °C; the minimum temperature of the coldest month ranged from 0 to 10 °C, with an optimal value around 7 °C; the annual average temperature ranged from 14 to 22 °C, with an optimal value around 16 °C; temperature seasonality ranged from 0 to 650 °C, with an optimal value around 400 °C; and the precipitation of the driest season ranged from 0 to 140 mm, with an optimal value around 50 mm.

### 3.3. Potential Distribution of Suitable Habitat for S. suberectus in the Current Period

**Figure 4:**
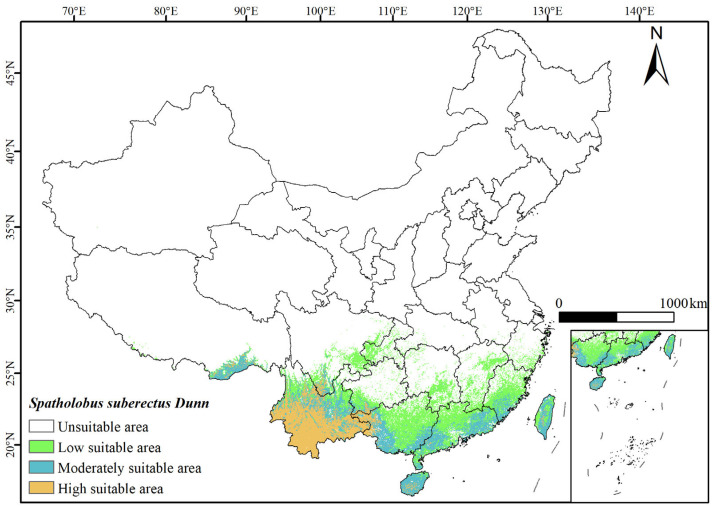
Predicted results of the ensemble model for *S. suberectus* in the current period. Yellow dots are data distribution points.

Based on the results of the combined Biomod2 model prediction (Figure 4), it was found that under the current scenario, the potential distribution of *S. suberectus* was primarily concentrated in the southern and southeastern coastal regions of China, mostly within tropical monsoon and subtropical monsoon climate zones. These areas are typically characterized by high temperatures, abundant precipitation, ample sunlight, and high humidity. The high-suitability habitat was mainly distributed in Yunnan Province, western Guangxi, southern Sichuan, and with scattered occurrences in Taiwan, southern Guizhou, eastern Guangdong, Central Hainan, southern Fujian, southeast Tibet, and western Chongqing, covering an area of 225.52 × 10^3^ km^2^. The medium-suitability habitat was mainly distributed in southeast Tibet, Taiwan, Hainan Province, southern Fujian, Yunnan Province, western Guangxi, the border areas between Guangxi and Guangdong, and eastern Guangdong, with scattered occurrences in Jiangxi Province, Hunan Province, southern Guizhou, and western Chongqing, covering an area of 285.93 × 10^3^ km^2^. The low-suitability habitat was found in southern China and the southeastern coastal areas, covering an area of 493.97 × 10^3^ km^2^.

### 3.4. Potential Distribution of Suitable Habitat for S. suberectus in Future Periods

**Figure 5:**
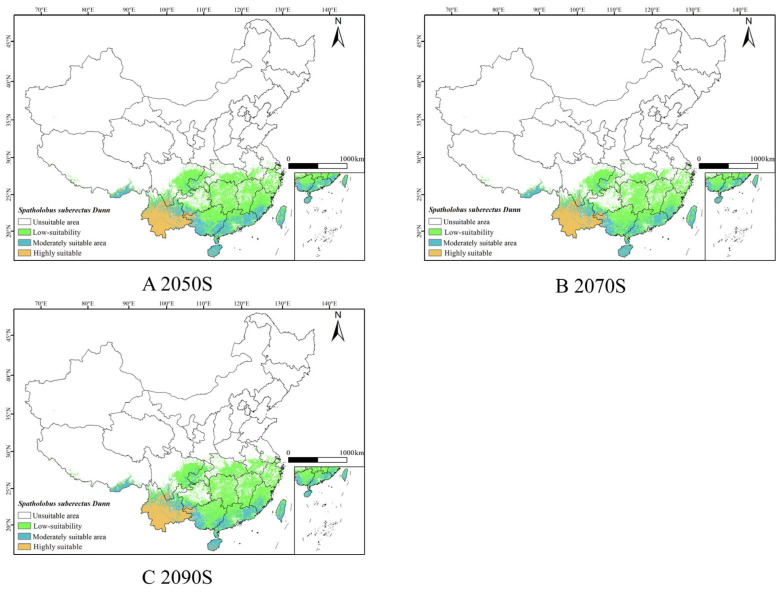
Predicted results of the ensemble model for *S. suberectus* in the 2050s, 2070s, and 2090s under the SSP1-2.6 scenario.

**Figure 6:**
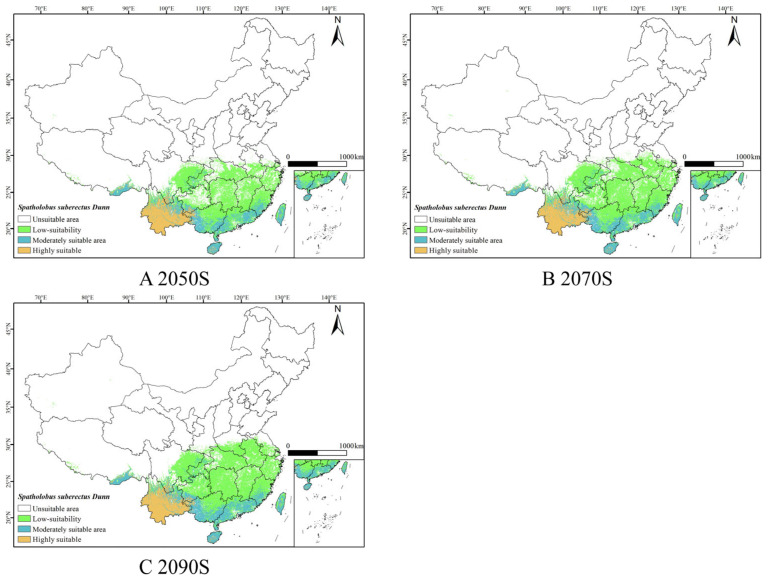
Predicted results of the ensemble model for *S. suberectus* in the 2050s, 2070s, and 2090s under the SSP2-4.5 scenario.

**Figure 7:**
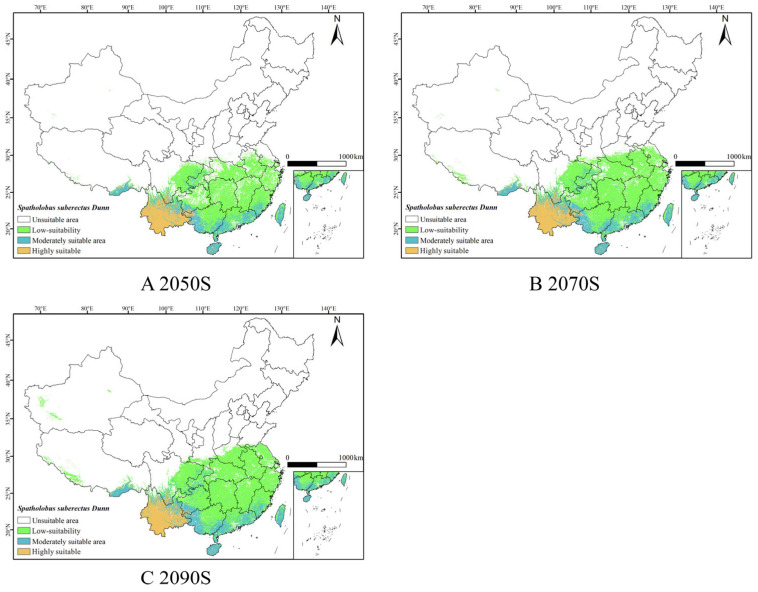
Predicted results of the ensemble model for *S. suberectus* in the 2050s, 2070s, and 2090s under the SSP5-8.5 scenario.

Under the SSP1-2.6, SSP2-4.5 and SSP5-8.5 scenarios, based on the combined model predictions for the 2050s, 2070s, and 2090s (Figure 5, Figure 6 and Figure 7), it was observed that the suitable habitat for *S. suberectus* would change in the future. In the future, the high-suitability habitat for *S. suberectus* would still be concentrated in Yunnan Province, where the temperature variation is small and precipitation is abundant, making it suitable for its growth. The following paragraphs describe the scenarios compared to the current period.

Under the SSP1-2.6 scenario, the increase in the low-suitability habitat is mainly concentrated in Sichuan Province, Hunan Province, Jiangxi Province, and Zhejiang Province, which is the higher in the 2090s by 533.11 km^2^; however, there is a reduction in the low-suitability areas in some regions, such as Yunnan and Hainan. The main distribution of the medium-suitability habitat is in Fujian, Guangxi, and Guangdong Provinces, which would increase by 77.82 × 10^3^ km^2^ in the 2050s; the high-suitability habitat would remain similar to the current distribution.

Under the SSP2-4.5 scenario, the distribution of the low-suitability habitat increased significantly, with Shanxi, Henan, and Jiangsu Provinces added. The medium-suitability habitat in Sichuan and Guangxi would increase; the high-suitability habitat showed no significant fluctuation.

Under the SSP5-8.5 scenario, the low-suitability area as a whole expands northward to Henan Province, Anhui Province, and Jiangsu Province. The distribution of moderately suitable areas in Taiwan, Guizhou Province, and Chongqing has increased, while the highly suitable areas in Guangxi have decreased slightly, but the overall distribution has increased (Table A1).

### 3.5. Area Changes of Suitable Habitat for S. suberectus in Future Periods

The distribution patterns of suitable habitat for *S. suberectus* in the current and future periods were compared and analyzed to evaluate the changes in suitable habitat area and the expansion and contraction of suitable habitat. Under the three future periods and three scenarios, the main expansion areas included Hunan Province, Jiangxi Province, Zhejiang Province, Shaanxi Province, Henan Province, and Jiangsu Province, with the expansion area peaking in the 2090s under the SSP5-8.5 scenario at 1290.31 × 10^3^ km^2^. The area of the suitable area of chicken blood vine shrank in some areas, and the main shrinkage area was Guangxi Province under the SSP5-8.5 scenario. Conversely, the suitable habitat area contracted in certain regions, particularly in Guangxi with the SSP5-8.5 scenario.

### 3.6. Changes in the Suitable Habitat Centroid for S. suberectus in the Future

**Figure 8:**
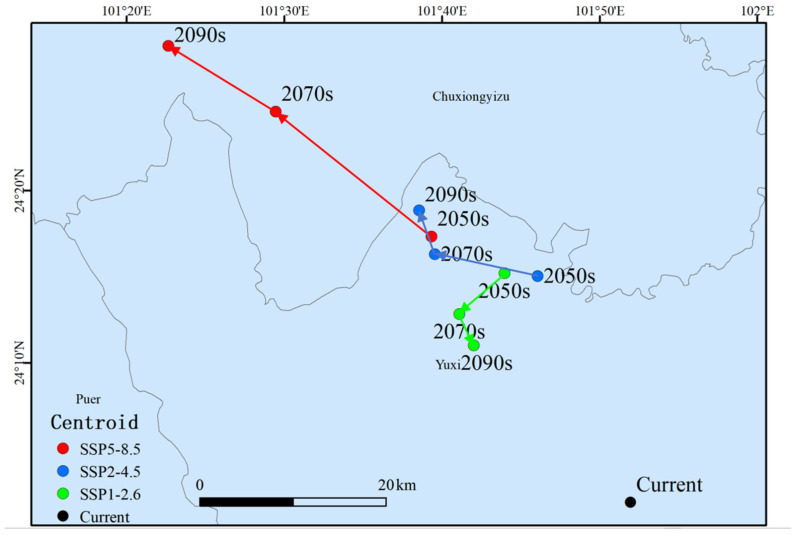
Changes in the centroid of the highly suitable habitat.

Under the SSP1-2.6, SSP2-4.5, and SSP5-8.5 scenarios, the changes in the centroid of the high-suitability habitat for *S. suberectus* were analyzed by combining the centroid shift maps (Figure 8). Over time, by the 2090s, the centroid of the high-suitability habitat generally shifted northwestward, followed by a northeastward shift. The centroid remained in Yunnan Province throughout all periods. In the current period, the high-suitability centroid was located in Yuxi Pingdian Township, Yunnan Province, with coordinates of 24.04° N, 101.87° E. In the SSP2-4.5 scenario, the centroids of the 2050s, 2070s, and 2090s were moved to Laochang Township, Yuxi, Yunnan, with coordinates of 24.26° N, 101.77° E, 24.28° N, 101.66° E and 24.32° N and 101.64° E, respectively. In the SSP5-8.5 scenario, the center of mass in the 2050s moved to Laochang Township, Yuxi, Yunnan Province, with coordinates 24.29° N, 101.66° E, the center of mass in the 2070s moved to Ainishan Township, Shuangbai County, Yunnan Province, with coordinates 24.41° N, 101.49° E, and the center of mass in the 2090s moved to Ejia Township, Shuangbai County, Yunnan Province, with coordinates of 24.47° N, 101.37° E.

## 4. Discussion

### 4.1. Evaluation of the Combined Model Performance

Currently, many studies tend to use single models to predict species distributions, but this approach carries the risk of overfitting or underfitting. Research has shown that combining the predictions of single models can reduce the prediction uncertainty of individual models, which will significantly enhance the robustness of prediction outcomes [41]. Furthermore, numerous studies have demonstrated that ensemble models can provide more reliable and stable predictions of species habitat suitability [42,43]. Biomod2 is particularly advantageous, as it can fit and compare different models, facilitating model selection [44]. Therefore, based on model screening, this study selected 11 single models to participate in the construction and accuracy evaluation of the ensemble model. Ultimately, six models with excellent TSS evaluation metrics (>0.9) (RF, XGBOOST, GAM, GBM, GLM, and MARS) were chosen to construct the ensemble model for predicting the geographical distribution of *S. suberectus* in China under global climate change scenarios. Although the evaluation results of single models indicated that the average Kappa and TSS values of the RF and XGBOOST models were slightly higher than those of the final ensemble model, this does not imply that single models are superior to the ensemble model in all aspects. The core value of ensemble modeling lies in its integration of predictions from multiple models with different algorithmic principles and inherent biases. This effectively reduces predictive fluctuations and uncertainties arising from reliance on a single model structure or specific dataset. Consequently, when faced with complex environmental variables and future climate change scenarios, ensemble models generally provide more robust and reliable predictions of distribution patterns. Therefore, although individual single models may perform exceptionally well on the training dataset, the ensemble model exhibits significant advantages in generalization ability and predictive stability, which is particularly critical for predicting the potential distribution of species under future climate change.

Overall, Biomod2, by integrating multiple models, can provide more robust prediction results even with relatively limited input distribution data [37,45,46]. The results of this study can provide a theoretical foundation for the conservation and utilization of *S. suberectus* in the future. Furthermore, this study mainly focuses on the influence of climatic factors (such as temperature and precipitation) on the distribution of *S. suberectu*. However, in the future, species distribution may also be significantly affected by non-climatic factors, such as land-use changes, human activity interference, soil properties, etc. [47,48]. Urbanization and deforestation may lead to habitat fragmentation or loss, resulting in actual distribution ranges being smaller than those predicted by climate models. For example, certain areas, although climatically suitable, may become unavailable for *Spatholobus suberectus* due to land development. Additionally, harvesting for traditional medicine may exert direct pressure on local populations, and even in climatically suitable regions, overharvesting could lead to population decline. *Spatholobus suberectus* also has specific requirements for soil conditions (e.g., pH, nutrient content, and drainage), which may restrict root development in karst regions or nutrient-poor lateritic soils, rendering climatically suitable areas uninhabitable in practice. Current models, based solely on climatic factors, may produce overly optimistic predictions. Therefore, future studies should integrate high-resolution land-use data to quantify habitat fragmentation effects or employ hybrid modeling approaches (e.g., mechanistic models coupling climate and soil moisture) to correct biases. Further integration of these non-climatic factors will enhance the comprehensiveness and accuracy of model predictions.

### 4.2. Influence of Environmental Factors on the Geographical Distribution of S. suberectus

This study investigates the environmental factors influencing the distribution of *S. suberectus*, with the contribution rate results indicating that the environmental factors mainly affecting the potential suitable area distribution of *S. suberectus* during three periods were temperature factors (such as the mean temperature of the coldest quarter, the minimum temperature of the coldest month, the annual mean temperature, and temperature seasonality) and precipitation factors (such as the precipitation during the driest season). This is mainly because *S. suberectus* grows in tropical and subtropical regions, where the warm and humid climate provides optimal conditions for temperature and precipitation for its growth. Temperature is one of the most critical environmental factors in plant growth and development, exerting multifaceted effects on plants. Research has shown that moderate temperatures can more effectively induce the accumulation of secondary metabolites (including phenolic and flavonoid compounds) in plants [49]. Drought and heat stress have adverse effects on plant physiological processes, leading to oxidative stress, cellular damage, reduced photosynthesis, and decreased productivity, which, in severe cases, can result in cell death [50]. Cold temperatures as low as −10 °C can effectively break dormancy in temperate woody plants [51]. At the same time, the results of this study are consistent with previous findings: precipitation plays a crucial role in shaping plant species distributions in mountainous ecosystems [52]. The optimal precipitation for *S. suberectus* occurs during the driest season at approximately 50 mm; both excessively high and low precipitation levels are detrimental to its growth and distribution. For *S. suberectus*, a suitable temperature is particularly critical for its growth, as it is highly sensitive to temperature. In this study, the contribution rate of temperature factors among environmental variables is 62.62%. In model predictions, the mean temperature of the coldest quarter contributes the most to the overall model. The response curve shows that when the mean temperature of the coldest quarter approaches 10 °C, its impact on the suitable growth regions of *S. suberectus* is maximal, promoting growth. When the minimum temperature of the coldest month is between 0 and 10 °C, growth is still promoted. This is because when plants are exposed to cold stress, they can gain tolerance to subsequent freezing conditions by temporarily being exposed to cold but non-freezing temperatures [53]. Additionally, key transcription factors within the plant help balance growth and stress tolerance [54]. Moreover, *S. suberectus* contains selenium, which aids in maintaining photosynthesis under temperature stress, enhancing the plant’s antioxidant capacity and mitigating oxidative damage under low temperatures [55]. When the mean temperature of the coldest quarter exceeds 0 °C but remains below 10 °C, or when the minimum temperature of the coldest month is below 0 °C, the suitability of *S. suberectus* for growth declines, and growth is inhibited. This indicates that *S. suberectus* has relatively low cold tolerance. Tolerance is one of the types of resistance responses plants exhibit to environmental challenges [56], and the response depends on the type and intensity of the environmental stress. One of the most prominent challenges is temperature variation, which significantly impacts the physiological and biochemical processes of plants [57,58]. In this study, *S. suberectus* is widely distributed in southern China, typically growing in tropical monsoon and subtropical monsoon climates. In warm climates, its growth rate is rapid. The results show that an annual mean temperature of around 16 °C is the optimal temperature for *S. suberectus* survival. From the results, it is clear that temperature factors are the dominant influence on the growth process of *S. suberectus*, and under the context of global warming, temperature changes will have profound effects on the growth and distribution of *S. suberectus*.

### 4.3. Future Changes in the Potential Suitable Distribution Areas of S. suberectus

This study employs the Biomod2 ensemble modeling framework to project dynamic changes in the potential suitable habitats of *Spatholobus suberectus* across China under three future climate scenarios. As a thermophilic and hygrophilous species, *S. suberectus* primarily inhabits sparse forests, shrublands, slopes, and valleys at elevations of 800–1700 m [6]. Under current climatic conditions, its distribution is concentrated in southern and southeastern coastal China, predominantly within tropical and subtropical monsoon climate zones. Empirical evidence indicates that without substantial greenhouse gas mitigation, global surface temperatures will rise by ≥2.1 °C by 2100, while the global mean sea level is projected to increase by 0.28–0.55 m by 2100 even under the lowest-emission scenario (SSP1-2.6) [59]. Elevation exerts a significant influence on the species’ distributional response under climate change. Rising temperatures are projected to drive upward shifts in its optimal elevational range, consistent with documented patterns of montane plants migrating to higher elevations to track thermally suitable conditions [60]. Although temperature typically declines with increasing altitude, high-elevation zones under global warming exhibit reduced frost frequency, enhancing the thermal suitability for *S. suberectus* [61]. For instance, in southwestern China, populations historically distributed at 800–1700 m may expand to 1700–2500 m, while habitat suitability at lower elevations (<800 m) may decline due to thermal stress.

Future temperature increases and sea-level rise will substantially alter distribution patterns. Under the SSP1-2.6, SSP2-4.5, and SSP5-8.5 scenarios, the centroids of highly suitable habitats remain anchored in Yunnan Province but exhibit divergent trajectories: a minor southward shift toward Yuxi under SSP1-2.6 versus northwestward shifts toward Shuangbai County under SSP2-4.5 and SSP5-8.5. This directional pattern corresponds to established biogeographic principles of poleward migration under warming [62,63], validating the Biomod2 model’s reliability. The pronounced northwestward shift under SSP5-8.5 likely reflects adaptive responses to arid thermal stress through seeking habitats with optimal hydrothermal equilibrium [64]. Scenario-specific analyses reveal distinct dynamics: Under SSP1-2.6 (low radiative forcing), suitable habitats exhibit moderate expansion with low-suitability areas extending northward to Sichuan, Hunan, and Jiangxi, peaking in the 2090s—consistent with extended growing seasons and positive responses of southwestern high-elevation vegetation [65]. For SSP2-4.5 (intermediate forcing), medium-suitability areas expand in Sichuan and Guangxi while highly suitable habitats remain stable, aligning with studies showing suppressed habitat suitability where summer temperatures exceed thermal tolerance thresholds in eastern China [64]. Under SSP5-8.5 (high radiative forcing), low-suitability areas shift northward to Henan, Anhui, and Jiangsu with maximal expansion in the 2090s, while highly suitable habitats in the Guangxi contract significantly—corresponding to projected heatwaves (>25 °C) exceeding the species’ thermal stress threshold (optimal growth ≤22 °C) [66]. Climate change disproportionately enhances habitat suitability in southwestern China (Yunnan and Guizhou), where future warming shifts temperatures toward the species’ optimum. The region’s warm, humid climate and complex topography provide substantial habitat heterogeneity, while temperature increases may extend growing seasons at higher elevations and enhance plateau productivity [67]. Conversely, South China (Guangxi and Guangdong) exhibits marked degradation of highly suitable habitats under SSP5-8.5 due to synergistic stressors: extreme summer temperatures (>25 °C) and precipitation variability. This high-temperature–drought co-stress induced contraction mirror vulnerability patterns in global tropical–subtropical transition zones [18]. Collectively, multi-scenario projections indicate the net expansion of suitable habitats, with Yunnan persisting as the core high-suitability area. These findings provide critical insights for conservation planning and the sustainable utilization of *S. suberectus*.

### 4.4. Optimization of Cultivation Zoning for S. suberectus in China Under Climate Change Scenarios

*S. suberectus* possesses significant pharmacological value. Due to its slow growth in the wild and overharvesting, its natural resources are becoming increasingly scarce. To meet the growing medicinal demand, it is essential to implement scientific cultivation planning, optimize production regions, and adapt to climate change for rational resource development. The results of this study indicate that in the three scenarios and the future three periods, the contraction areas are mainly concentrated in South China. In these regions, future conservation strategies should prioritize reducing reliance on wild resources by transitioning to cultivated plantations, protecting existing populations, and preventing habitat fragmentation [68]. The expansion areas are mainly concentrated in southwest, central, and East China. Notably, in the three scenarios and the future three periods, the center of gravity of the high-suitability areas will always remain in Yunnan Province. To mitigate the negative impact of climate change on the kudzu vine in the future, Yunnan should be designated as a core protection zone, with measures implemented to curb overharvesting and promote sustainable resource utilization. Previous studies have demonstrated that climate warming significantly influences species distribution, with the rate of warming positively correlated to the speed of range shifts [69]. In response to the rapid climatic adaptation of species, a dynamic monitoring network should be established to mitigate cultivation risks [70]. Geographic Information System (GIS) technology can be employed to update suitable habitat boundaries in real time, optimize harvesting zones, and align management strategies with centroid migration trajectories [71]. In conclusion, given the sustained impacts of climate change on the distribution of medicinal plant resources, it is imperative to implement rational planning for future production zones of *S. suberectus.*

## 5. Conclusions

This study predicts and analyzes the future expansion and contraction of the potential suitable distribution areas of *Spatholobus suberectus* in China under the influence of climate and environmental data changes and puts forward some suggestions on origin planning using the Biomod2 ensemble model. Under the SSP1-2.6 scenario, the distribution of *Spatholobus suberectus* in the future three periods shows a trend of first expansion and then contraction. In the 2070s, the suitable habitat area has significantly shrunk, with the contracted areas mainly concentrated in the southwest and central China regions. Under the SSP2-4.5 and SSP5-8.5 scenarios, the distribution of *Spatholobus suberectus* in the future three periods shows an expansion trend. The expanded areas are mainly concentrated in the southwest, central, and eastern China regions, with a small part located in the western Xinjiang and western Tibet regions. Compared with the current suitable habitat distribution, the suitable habitat range of *Spatholobus suberectus* in different future periods under the three scenarios is still mainly concentrated in the tropical monsoon climate and subtropical monsoon climate regions. The primary factors influencing the distribution of *S. suberectus* in China are climate factors, though topography and soil factors also play a synergistic role. Overall, climate change has a significant impact on the potential distribution areas of *Spatholobus suberectus* in the border areas of the southwest provinces and central China. Future development and utilization of *S. suberectus* in China should closely monitor the impacts of climate change while also preventing the future contraction of its suitable areas to ensure ecological balance and sustainable development.

## Figures and Tables

**Table 1:** Environmental variables.

Environment Variables	Variable	Unit
gm_lc_v3	Soil cover type	_
gm_ve_v2	Vegetation cover percentage	_
hf_v2geo1	Human footprint and activity impact index	_
annual_mean_uv-b	Annual average ultraviolet radiation	kWh/m^2^
elev	Altitude	m
slope	Slope	°
aspect	Aspect	_
bio01	Annual average temperature	°C
bio03	Isothermality	_
bio04	Temperature seasonality	_
bio05	Maximum temperature of the warmest month	°C
bio06	Minimum temperature of the coldest month	°C
bio09	Average temperature of the driest season	°C
bio11	Average temperature of the coldest season	°C
bio17	Precipitation of the driest season	mm
d1_clay	Clay content	cps
d1_cn_ratio	Carbon-to-nitrogen ratio	_
d1_elec_cond	Electrical conductivity	_
d1_ph_water	pH level	pH
d1_sand	Sand content	kg/m^3^
d1_swr	Soil moisture status	_
d1_total_n	Total nitrogen content	mg/L
d1_usda	Soil texture classification	_

**Table 2:** TSS and KAPPA evaluation criteria.

TSS	KAPPA	Evaluation Criteria
0 ≤ TSS ≤ 0.4	0 ≤ KAPPA ≤ 0.4	Lose
0.4 < TSS ≤ 0.55	0.4 < KAPPA ≤ 0.55	Normal
0.55 < TSS ≤ 0.7	0.55 < KAPPA ≤ 0.7	Good
0.7 < TSS ≤ 0.85	0.7 < KAPPA ≤ 0.85	Fine
0.85 < TSS	0.85 < KAPPA	Superb

## Data Availability

The data that support the findings of this study will be available in figshare at https://doi.org/10.6084/m9.figshare.28559795 (accessed on 7 August 2024) (GBIF, https://doi.org/10.15468/dl.hy9gcv, accessed on 7 August 2024).

## Appendix A

**Table A1:** Suitable habitat area of *S. suberectus* in different periods.

Epoch	Low-Suitability Habitat (×10^3^ km^2^)	Moderate-Suitability Habitat (×10^3^ km^2^)	High-Suitability Habitat (×10^3^ km^2^)
Current	493.97	285.93	22.552
SSP1-2.6 2050S	948.17	363.75	258.05
SSP1-2.6 2070S	911.89	320.15	247.58
SSP1-2.6 2090S	1027.08	334.02	245.95
SSP2-4.5 2050S	1043.92	360.41	258.66
SSP2-4.5 2070S	1207.60	386.84	249.91
SSP2-4.5 2090S	1331.57	423.99	258.78
SSP5-8.5 2050S	1194.47	353.42	256.30
SSP5-8.5 2070S	1456.96	403.35	262.96
SSP5-8.5 2090S	1598.92	434.87	261.94
